# Acetazolamide Treatment Prevents Redistribution of Astrocyte Aquaporin 4 after Murine Traumatic Brain Injury

**DOI:** 10.1155/2019/2831501

**Published:** 2019-05-02

**Authors:** Nancy K. Glober, Shane Sprague, Sadiya Ahmad, Katherine G. Mayfield, Lauren M. Fletcher, Murat H. Digicaylioglu, Naomi L. Sayre

**Affiliations:** ^1^Department of Emergency Medicine, Stanford University, Palo Alto, California, USA; ^2^Department of Neurosurgery, University of Texas Health Science Center at San Antonio, San Antonio, Texas, USA; ^3^South Texas Veteran's Health Care System, San Antonio, Texas, USA

## Abstract

After traumatic brain injury (TBI), multiple ongoing processes contribute to worsening and spreading of the primary injury to create a secondary injury. One major process involves disrupted fluid regulation to create vascular and cytotoxic edema in the affected area. Although understanding of factors that influence edema is incomplete, the astrocyte water channel Aquaporin 4 (AQP4) has been identified as an important mediator and therefore attractive drug target for edema prevention. The FDA-approved drug acetazolamide has been administered safely to patients for years in the United States. To test whether acetazolamide altered AQP4 function after TBI, we utilized* in vitro *and* in vivo *models of TBI. Our results suggest that AQP4 localization is altered after TBI, similar to previously published reports. Treatment with acetazolamide prevented AQP4 reorganization, both in human astrocyte* in vitro *and in mice* in vivo. *Moreover, acetazolamide eliminated cytotoxic edema in our* in vivo *mouse TBI model. Our results suggest a possible clinical role for acetazolamide in the treatment of TBI.

## 1. Introduction

Two types of edema can occur after a brain injury. Vasogenic edema is caused by bulk fluid retention in the brain, resulting in swelling and increased intracranial pressure. Cytotoxic edema is caused by fluid retention in individual cells, resulting in cellular necrosis. Edema is influenced by Aquaporins, a family of transmembrane tetramer proteins that regulate water homeostasis in cells throughout the body [1]. AQP4 is expressed in astrocytes and is the most common Aquaporin in the central nervous system [2]. The importance of AQP4 in paravascular fluid circulation was demonstrated in the brain, when loss of AQP4 resulted in decreased flux of cerebrospinal fluid through brain parenchyma [3]. Termed the “glymphatic system”, this paravascular circulation relies on movement of fluid across astrocytes through AQP4. Given these findings, disruption of AQP4 function would have grave consequences for brain fluid homeostasis.

TBI disrupts AQP4 function. Normally, AQP4 is localized specifically on perivascular endfeet processes of astrocytes at the blood brain barrier [2, 4]. Ren et al. found that TBI causes redistribution of AQP4 away from endfeet and into the fine astrocyte processes after TBI [5]. Different groups have shown both increased [6–9] and decreased [10–13] AQP4 expression after TBI. These disparate findings may be due to a dynamic process of AQP4 reorganization—AQP4 expression increases in perivascular endfeet as edema resolved in a rat model of TBI [14, 15].

Further clarification is needed in understanding how AQP4 influences edema. Findings have been contradictory as to whether AQP4 promotes or eliminates edema, and whether AQP4 is beneficial or harmful to recovery after brain damage. Overexpression of AQP4 is detrimental to survival after water intoxication [16], and AQP4 deletion improves survival after water intoxication as well as ischemic stroke [17]. Similarly, loss of AQP4 improves survival after bacterial meningitis [18]. In contrast, loss of AQP4 was harmful in multiple models of brain edema [19], and chronic brain injury was aggravated in AQP4 null mice after focal cerebral injury [20]. Regardless of the exact role, these sometimes contradictory studies indicate the importance of AQP4 in the pathogenesis of edema and suggest that AQP4 is an ideal drug target in combatting brain edema.

We chose to target AQP4 using acetazolamide. Acetazolamide, an inhibitor of sulfonamide carbonic anhydrase (CA), blocks AQP4 water conductance through liposomes [21] and prevents AQP4 upregulation after TBI [22]. Acetazolamide is a safe, FDA-approved drug, currently used as a diuretic and altitude sickness prophylactic. We were able to block oxygen glucose deprivation (OGD) induced changes in AQP4 distribution* in vitro* with acetazolamide. Similarly, we found that acetazolamide prevented TBI-induced reorganization of AQP4 away from astrocyte endfeet* in vivo*. Mice subjected to TBI and treated with acetazolamide showed less cytotoxic edema than their untreated counterparts. Based on our experiments, the localization of AQP4 in astrocytes plays a fundamental role in cytotoxic edema and suggests that targeting AQP4 localization with acetazolamide could be a viable clinical treatment for cytotoxic edema after TBI.

## 2. Results

### 2.1. Aquaporin 4 Cellular Localization Is Altered in a Cultured TBI Model

While TBI is the result of an initial mechanical injury, several ongoing processes contribute to secondary spread of damage. Notably, shearing of blood vessels and disruption of the blood brain barrier (BBB) contribute to decreased circulation of nutrients, metabolic crisis, and altered water homeostasis in the affected area [23–27]. To duplicate these events in culture, we utilized an* in vitro *stretch and OGD model, similar to previously published models [28–31]. Primary rat astrocytes were cultured on a 2 mm silastic membrane and then subjected to a single stretch injury by deforming the membrane by 30% for 5 seconds using a Flexcell Vacuum Tension System. Astrocytes were subsequently subjected to metabolic stress by OGD for 6-48 hours. We tested the effect of our cultured TBI model on AQP4 expression and localization in primary rat astrocytes.

**Figure 1:**
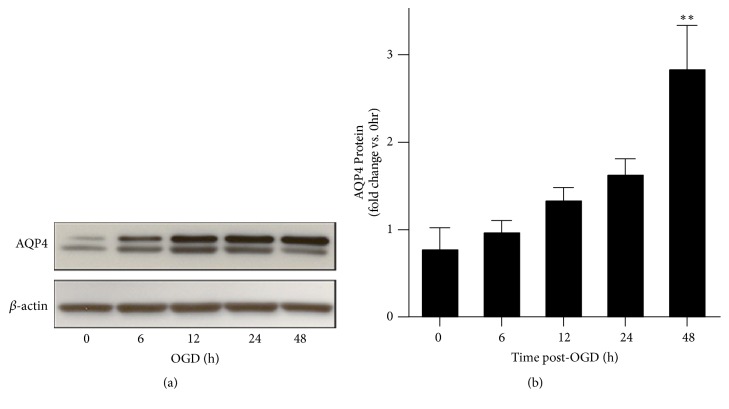
*AQP4 total protein levels increase in an in vitro trauma model*. Primary rat astrocytes were subjected to a single stretch injury and then OGD for 0, 6, 12, 24, and 48 hours. (a) Representative western blot for AQP4 and an actin loading control. (b) Densitometry measurements from 4 separate independent western blots show increased AQP4 protein expression at 48 hours of OGD.* Results are averages ± SEM. Significant differences were determined with one-way ANOVA followed by Tukey's HSD test. ∗∗p<0.01.*

**Figure 2:**
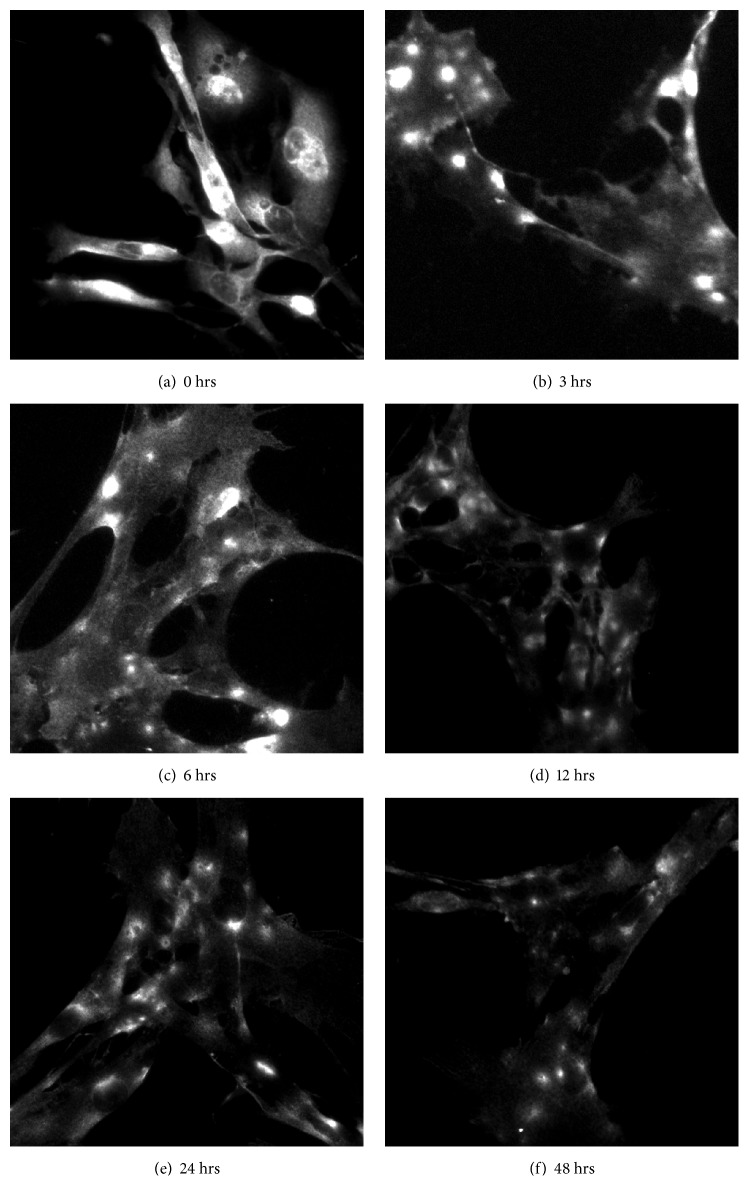
*AQP4 reorganizes puncta in an in vitro trauma model*. AQP4 distribution was measured via immunocytochemistry in primary rat astrocytes subjected shear stress and then to OGD for (a-f) 0, 6, 12, 24, and 48 hours (n=3/group, 3 independent experiments).

To determine whether AQP4 protein expression was altered, we measured total AQP4 levels via SDS-PAGE and western blotting. We found that total AQP4 protein expression did not significantly increase until 48 hours of OGD had elapsed (Figures 1(a) and 1(b)) similar to previous studies (Sturdivant et al., 2016). Other labs have reported that disrupted localization and polarization of AQP4 contributes to edema phenotype* in vivo* [4, 5]. Therefore, we tested whether AQP4 localization is altered in our* in vitro *TBI model using immunocytochemistry to stain plasma membrane and intracellular AQP4. We found that AQP4 localization changed from a diffuse, plasma membrane distribution to a discrete and punctate localization upon OGD in as little as 3 hours and was maintained for up to 48 hours (Figure 2). We similarly obtained higher resolution images of cells after OGD and observe that AQP4 seems to reorganize from a plasma membrane expression to a more endosomal pattern of expression (Figure S3). Moreover, we tested whether stretching* per se* elicited alterations in expression patterns. In the absence of OGD, stretching failed to cause changes in AQP4 localization (Figure S4). We did note possible differential expression of the M1 and M23 isoforms over time, specifically that M23 peaked at 12 hours and was more dominant in the control than in the OGD model cells (Figure 1), suggesting a possible mechanism for regulation in OGD.

### 2.2. Acetazolamide Prevents Aquaporin 4 Redistribution in a Cultured TBI Model

**Figure 3:**
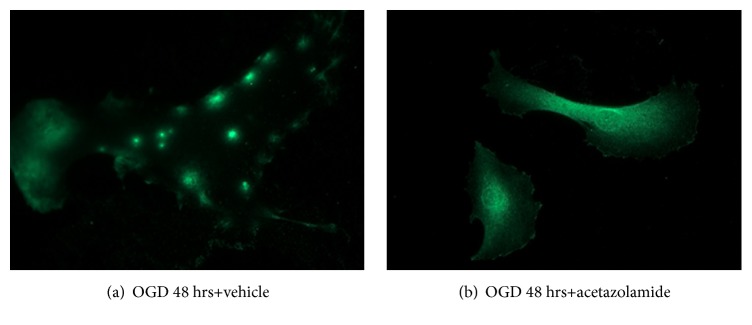
*Acetazolamide treatment prevents AQP4 reorganization in human astrocytes*. Astrocytes were treated with (a) vehicle or (b) acetazolamide (0.45 *μ*m) at the onset of OGD. At 48 hours, astrocytes were fixed and subjected to APQ4 immunocytochemistry (n=3/group, 3 independent experiments).

Acetazolamide targets AQP4 [21, 22]. We tested whether acetazolamide altered AQP4 localization in our* in vitro *TBI model. Here, we treated human (rather than rat) astrocytes, with the rationale that an effect observed in human cells would more readily translate into human treatments. As with rat astrocytes, human astrocytes showed a reorganization of AQP4; however, this reorganization occurred at 24 hours of OGD (not shown). We treated human astrocytes with acetazolamide (0.2 mg/mL in DMSO) and then subjected them to our* in vitro* TBI model (Figure 3). Acetazolamide treatment prevented the punctate aggregation of AQP4 after OGD (Figure 3(b)), unlike vehicle treated cells (Figure 3(a)). These results suggest that acetazolamide alters AQP4 distribution and possibly function in humans.

### 2.3. Aquaporin 4 Localization Is Disrupted after TBI In Vivo

**Figure 4:**
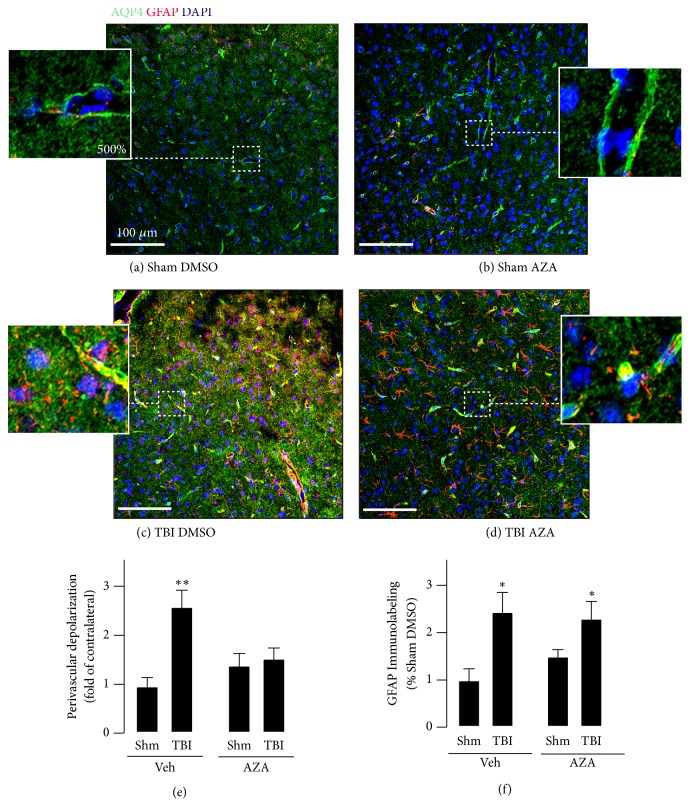
*Acetazolamide treatment prevents TBI-induced AQP4 depolarization*. Mice were subjected to sham surgery or closed-cortical impact surgery, and were treated 30 minutes after surgery with either DMSO vehicle (Veh) or acetazolamide (15 mg/kg, AZA). Mice were sacrificed at 3 days after injury. Immunohistochemical labeling for AQP4 (green), GFAP (red), and DAPI (blue) was performed, and representative confocal images are shown in (a) sham-DMSO-treated mice, (b) sham-acetazolamide (AZA) treated mice, (c) TBI-DMSO-treated mice, and (d) TBI-AZA treated mice. Inset images show areas of increased magnification. (e) Histogram plot of perivascular depolarization was measured as described in the* methods *section (n=6 mice/group; *∗∗*p<0.01 versus all other groups) (f) Histogram plot of GFAP immunolabeling expressed as a percentage of sham-DMSO-treated mice (n=6 mice/group; *∗*p<0.05 versus sham-DMSO or sham-AZA treated mice).* Significance was measured using ANOVA followed by Tukey's HSD test.*

It has been shown that TBI elicits a change in localization of astrocyte AQP4 in mice by 3 days after injury [5]. They found that normally, AQP4 maintains a polarized expression that is concentrated to astrocyte endfeet in the perivascular domain and that this polarization is lost upon TBI. To test whether AQP4 reorganization occurs in our* in vivo* closed-cortical injury model, we subjected mice to a single and moderate closed-cortical impact and treated with acetazolamide or vehicle at 30 minutes after injury. We tested localization of AQP4 near the injury site using immunohistochemistry of tissue sections harvested 3 days after TBI. In sham-operated mice, we found that AQP4 was concentrated into structures morphologically consistent with astrocyte endfeet, which wrap around vessels to maintain the BBB (Figure 4(a)). In mice subjected to TBI, we found disrupted AQP4 localization in vehicle treated animals, such that increased levels of AQP4 were observed in the fine astrocytic processes, indicating depolarization of AQP4 away from end feet towards astrocyte fine processes and cell bodies (Figures 4(c) and 4(e)). We similarly measured the ratio of M1 AQP4 isoform to M23 and found a significant increase in M1:M23 ratio in the cortex of control mice after TBI. This increase was alleviated in mice treated with AZA (Figure S5).

### 2.4. Acetazolamide Prevents Aquaporin 4 Relocalization In Vivo after TBI

Next, we tested whether acetazolamide treatment could affect AQP4 localization* in vivo. *Acetazolamide did not alter AQP4 organization in sham-operated animals, (Figure 4(b)); however, acetazolamide treatment after TBI prevented the reorganization of AQP4 away from endfeet (Figures 4(d) and 4(e)). To test whether acetazolamide altered astrocyte activation, we measured expression of the astrocyte marker glial fibrillary acidic protein (GFAP), which becomes upregulated after injury [32]. We found that TBI increased cortical expression of GFAP regardless of acetazolamide treatment (Figure 4(f)). Altogether, these results suggest that acetazolamide prevents reorganization of AQP4 but does not prevent astrocyte activation.

### 2.5. Acetazolamide Prevents Cytotoxic Edema In Vivo after TBI

**Figure 5:**
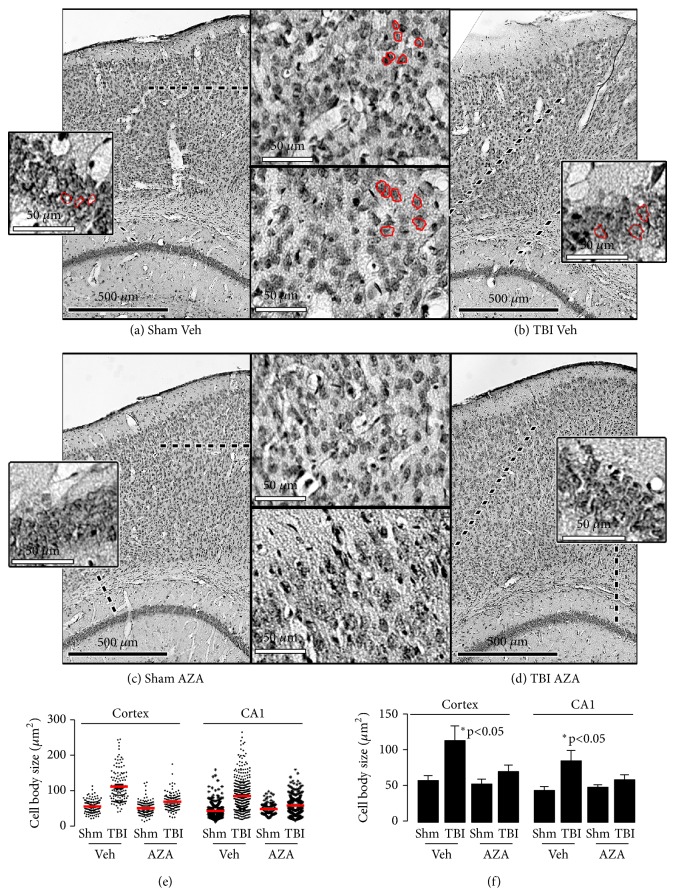
*Acetazolamide reduced cytotoxic edema in cell bodies within the cortex and hippocampus of mice subjected to TBI*. Mice (n=6/group) were subjected to sham surgery or closed-cortical impact surgery, and were treated 30 minutes after surgery with either DMSO vehicle (Veh) or acetazolamide (15 mg/kg, AZA). At 1-day after injury, mice were sacrificed. Representative images of Nissl-stained sections are shown of mice subjected to (a) sham surgery and vehicle treatment, (b) TBI with vehicle treatment, (c) sham surgery with acetazolamide, or (d) TBI with acetazolamide treatment. Inset boxes detail higher magnification views of the indicated regions in either the CA1 region of the hippocampus or the cortex. Representative cells have been circled in red. Cell body sizes were measured and were plotted either as (e) a dot plot, with the mean indicated in red or as (f) a histogram plot. *∗Significance was measured using ANOVA followed by Tukey's HSD test.*

Given that acetazolamide prevents the reorganization of AQP4 after TBI, we measured the effect of treatment on cytotoxic edema in our closed-cortical injury model at 24 hours after TBI. Vasogenic edema was tested by measuring water content of brains, similar to previously published reports [32–34]. Given that our model involves mild concussive injury, we did not expect to find any alterations in vasogenic edema. Indeed, we did not observe any significant changes in overall brain water content after mild TBI in our mouse model. We similarly did not measure any effect of acetazolamide on total brain water content (not shown). Next, we measured cytotoxic edema via Nissl staining of frozen tissue sections, similar to previously published studies [32] (Figure 5). We found that cell bodies of neurons within the cortex (Figures 5(a), 5(c), and 5(e)) and CA1 region of the hippocampus (Figures 5(b), 5(d), and 5(f)) of mice subjected to TBI significantly increased in size compared to sham, indicating cytotoxic edema. In contrast, mice treated with acetazolamide had cell bodies comparable to sham treated mice. Altogether, these results indicate that acetazolamide diminished cytotoxic edema after TBI.

## 3. Discussion

We observed altered distribution of AQP4 in our* in vitro* TBI model, when AQP4 changed from a diffuse membrane distribution to more punctate, localized clusters. Moreover, we confirmed previous* in vivo *results from Ren et al. [5] that found that TBI causes a redistribution of AQP4 away from perivascular endfeet in cultured astrocytes. Importantly, the reorganization of AQP4 could partially account for the disparate findings, in which some labs have found increased AQP4 after TBI but others have found decreased AQP4. The reorganization of AQP4 to different cellular compartments may render AQP4 more or less susceptible to extraction depending on the lysis buffer and therefore could be interpreted as altered global AQP4 protein expression.

After* in vitro* traumatic injury, we found AQP4 clustered, possibly into aggregates termed orthogonal arrays of particles (OAPs) or possibly into the endocytic system, although we did not extensively quantify this difference* in vitro*. Others have visualized OAPs of AQP4 via freeze-fracture electron microscopy [35], immunogold electron microscopy [36, 37], and super-resolution fluorescence microscopy [38]. It has been suggested that aggregates of AQP4 could be involved in water permeability [39], cellular adhesion [40], and cellular polarization [41], but the exact function of OAPs is poorly understood [42]. The shorter M23 isoform of AQP4 is thought to be responsible for assembly of OAPs and is expressed due to “leaky scanning” of the full-length M1 mRNA at a second start codon [43, 44]. We did note that there was likely differential expression of the M1 and M23 isoforms over time* in vitro *(Figure 1), specifically that M23 peaked at 12 hours and was more dominant in the control than in the OGD model cells, suggesting a possible mechanism for regulation in OGD. The clusters of AQP4 we observed formed early, consistent with our observation that M1 increased quickly in OGD conditions. We similarly examined the M1:M23 ratio* in vivo *after TBI. In the cortex, TBI induced an increase expression of M1, consistent with our* in vitro *data. M1 levels did not rise when TBI mice were treated with acetazolamide. While our information is insufficient to conclude whether OAPs have formed, it is known that the ratio of M1:M23 can alter the size and formation of OAPs; indeed M1 expression alone is insufficient to cause OAP formation, whereas M23 expression alone causes formation of larger OAPs, and combined expression causes intermediate sized OAPs [44]. Together, the data suggest that the rise in expression of M1 compared to M23 may have decreased OAP size and may be associated with decreased water permeability [45, 46]. This, in turn, might account for the decreased cytotoxic edema we observed. Further studies are needed to identify the dynamic ratio of M1/M23 during ischemic insult or TBI, and how the relationship between M1 and M23 changes with acetazolamide treatment.

Some studies have shown that AQP4-null mice demonstrate less cytotoxic edema after brain damage, suggesting the possibility that the absence of AQP4 on astrocytes helps prevent cytotoxic edema. However, we noted in the introduction that the effect of AQP4 is largely context dependent, at times aiding in the resolution of edema while at other times hampering it. Based on our results, we suggest that AQP4 is important to the interrelationship of vasogenic and cytotoxic edema. The putative dual role of AQP4 in resolving vasogenic edema while worsening cytotoxic edema could similarly account for the sometimes contradictory findings that AQP4 aggravates brain injury [16–18] but other times reduces injury [19, 20]. Depending on the model, either vasogenic or cytotoxic edema could independently contribute to morbidity. AQP4 may mitigate vasogenic edema at the expense of worsening cytotoxic edema. In our mild TBI model, AQP4 may prove beneficial particularly to cytotoxic edema because significant vasogenic edema was never a problem.

Our results suggest a potential clinical treatment for cytotoxic edema using acetazolamide. We discovered that acetazolamide prevented the reorganization of AQP4 in response to OGD or TBI. Tanimura et al. described the physical blockade of water by acetazolamide through AQP4* in vitro* in liposomes of AQP4 [21]. However, to our knowledge, no one has described the ability of acetazolamide to prevent the change in AQP4 localization* in vivo*. We observed that acetazolamide blocked the development of cytotoxic edema in both the cortex and the hippocampus and prevented the reorganization of AQP4 away from astrocyte endfeet. Based on previous studies, it was unsurprising to discover that acetazolamide could prevent cytotoxic edema, possibly by physical blockade of the AQP4 channel [21]. However, it was interesting to note that the redistribution of AQP4 was mitigated by acetazolamide. Moreover, acetazolamide caused the M1:M23 ratio to return to control levels, suggesting a possible mechanism by which edema and AQP4 distribution are regulated. If acetazolamide blocks water flow, then it suggests that flow of water through the membrane pore could itself trigger redistribution to less accessible compartments. Alternatively, acetazolamide might contribute to increased “leaky scanning” of AQP4 mRNA to alter M1:M23 ratios. However, further studies are needed to clarify the mechanism by which acetazolamide prevents redistribution of AQP4. Indeed, our experiments have not shown whether AQP4 is the direct target of acetazolamide or whether the targeting of AQP4 was the cause for resolution of cytotoxic edema. Future experiments would include testing the effect of acetazolamide on edema in the absence of AQP4, for instance, in AQP4 null mice.

Acetazolamide treatment appears beneficial in our mild TBI model and could be a useful treatment in reducing injury after mild TBIs or concussions. This could particularly be true in the cases of patients (such as athletes) who suffer from repeated mild or subconcussive injuries and are at greater risk of developing chronic traumatic encephalopathy with aging. A promising route of further exploration would test whether acetazolamide treatment after concussion prevents age-related declines in brain function.

Even though acetazolamide is promising in the treatment of cytotoxic edema, it is unclear whether it would be beneficial to patients with greater degrees of vasogenic edema. Ren and colleagues proposed that the reorganization of AQP4 was a mechanism to facilitate resolution of vasogenic edema [5]. Because our model does not exhibit significant vasogenic edema, we were unable to test this possibility. However, given that AQP4 seems to play differential roles in resolving different type of brain injury, it would not be surprising if acetazolamide has varying efficacy depending on the injury model.

Finally, we also measured astrocyte activation in the cortex by measuring expression of GFAP. We found that astrocyte activation appears unaffected by treatment with acetazolamide, as GFAP expression increased after TBI regardless of treatment. It is unclear whether the continued activation of astrocytes is indicative of continued expansion of secondary injury, as other factors besides edema could contribute to astrocyte activation, including metabolic disturbances [32], recurrent spreading depolarizations [47, 48], or immune modulation [49]. Nevertheless, based on our findings, acetazolamide could potentially be an effective and safe drug for treating TBI in humans to prevent pathological cytotoxic edema.

## 4. Materials and Methods

### 4.1. Materials

Unless otherwise noted, astrocytes were cultured in astrocyte media: DMEM (Life technologies, Austin TX) with 10% fetal calf serum (ATCC, VA). The following antibodies were utilized for studies: antiglial fibrillary acidic protein (Millipore AB5541, Darmstadt Germany, 1:500); antiaquaporin 4 (Millipore, AB3594, 1:200); anti-chicken alexa 568 (Thermofisher, A-11041, 1:200); anti-rabbit alexa 488 (Thermofisher, A-11008, 1:200). Acetazolamide was purchased from Sigma (St. Louis, MO).

### 4.2. Isolation of Primary Rat Astrocytes

Pregnant females Sprague Dawley rats (Charles Rivers Laboratories, MA) were euthanized by placing rats in deep anesthesia (with 5% isoflurane) followed by cervical dislocation. Embryonic day 17 (E17) rat embryos were harvested, and the embryonic cerebral cortices were isolated. To obtain astrocytes, cortices were manually chopped into 1-2 mm3 pieces and then were enzymatically digested with trypsin-EDTA (0.125%, Life Technologies) for 30 min at 37°C. Cells were mechanically dissociated by titration through a glass pipette 10 times, and then nondissociated tissue was filtered away with a 40 *μ*m cell strainer. Cells were collected via centrifugation (700Xg, 2 min) and then immediately cultured in astrocyte media at 37°C, 5% CO_2_, with weekly media changes until subconfluent. Immunocytochemisty confirmed that over 90% of the cells expressed the astrocyte marker GFAP (Figure S1). Astrocytes were used for experiments up to passage 5 and then were discarded.

### 4.3. Isolation of Primary Human Astrocytes

In collaboration with the Department of Neurosurgery (University of Texas Health Science Center, San Antonio TX), fresh tissue was donated from patients undergoing temporal lobe resection for epilepsy. All tissue-harvesting procedures were performed under approval from the University of Texas Health Science Center San Antonio Institutional Review Board with written patient consent. Investigators had no access to any identifiable information about the patients, other than the age and sex of the patient. Brain tissue was acutely dissected from adult neurosurgical patients, placed in a 50 mL conical tube and rinsed with chilled PBS/Penicillin/Streptomycin. Under the hood, the brain tissue was placed into a 60 mm dish with 3 mL of 0.25% trypsin and chopped into small pieces using a razor blade. The tissue was incubated at 37°C for 20 min. The digested cells were mechanically dissociated by titration and filtered through a 40 *μ*m cell strainer. Previous analysis of human astrocytes showed very low expression of other nonastrocytic neural cell markers [50].

### 4.4. In Vitro TBI Model

Astrocytes were cultured on 6-well BioFlex culture plates (Flexcell International, NC) to 70-80% confluence in astrocyte media. The evening before experimentation, media was switched to serum-free DMEM. Cells were subjected to a single stretch injury by fitting the BioFlex plates onto the Flexcell Tension System (Flexcell International), and then the membrane at the bottom of the plates was stretched one time for 5 sec to 30% elongation. To mimic metabolic disruptions that occur with loss of perfusion after TBI, media were immediately switched to serum-free, glucose free DMEM (Life Technologies), and astrocytes were allowed to recover in a hypoxic chamber (1% O_2_, 94% N_2_, 5% CO_2_) at 37°C.

### 4.5. Immunocytochemistry

Astrocytes were fixed with 4% paraformaldehyde (10 mins) and washed 3 times with PBS (5 mins). Cell membranes were solubilized with 0.5% triton-X 100 in PBS (5 mins), followed by additional 3 washes with PBS (5 mins). Nonspecific blocking was achieved by incubating with 5% bovine serum albumin (Sigma, MO) for 1.5 hours. Primary antibodies were incubated with astrocytes in blocking buffer overnight at 4°C. After washing 3 times with PBS (15 mins), astrocytes were incubated in secondary antibody in blocking buffer for 1 hour and then washed again with PBS (5 min). Slides were mounted with ProGold Antifade (Invitrogen, MA) before visualization. Cultured astrocytes were imaged via fluorescent microscopy on a Zeiss Axio Imager A1 upright microscope using a 20X EC-Plan NeoFluor 20X/0.5 NA objective. Specific labeling of AQP4 was confirmed by incubating the primary antibody with an AQP4 synthetic peptide before ICC and visualizing a loss of AQP4-specific signal (Figure S2)

### 4.6. Closed-Cortical Injury of Mice

Unless otherwise specified, rodents were maintained on a standard 12 hour light/dark cycle, fed standard rodent chow, and were housed up to 5 mice/cage in the University of Texas laboratory animal facility. All rodent procedures were carried out with approval from the University of Texas Health Science Center Institutional Animal Care and Use Committee, and protocols were utilized which were consistent with the NIH guide on the care and use of animal models. A pneumatic impact device was used to generate a moderate TBI (n=6/treatment group/experiment) at 4.5 m/s to 2.0 mm depth, with no disruption of the skull or dura mater. Three-month-old male C57BL/6 mice (Jackson laboratories) were anesthetized with isoflurane (3% induction, 1% maintenance) in 100% oxygen. A 5.0 mm stainless steel disc was surgically positioned on the skull with superglue on the right parietal bone between bregma and lambda over the somatosensory cortex. Apneic episodes after injury were timed. Scalp incisions were closed with superglue, antibiotic ointment (Neosporin-Johnson and Johnson, New Jersey USA) was applied, and mice were treated with a single injection of 300 *μ*L of subcutaneous lactated ringers for hydration and 0.05mg/kg subcutaneous buprenorphine for pain relief. Thirty minutes after injury, mice were treated with 15 mg/kg acetazolamide suspended in DMSO via intraperitoneal injection. Mice were allowed to wake to full sternal recumbency in a hydrated, warm rodent ICU chamber (approximately 30-45 minutes) and then were checked daily thereafter to ensure the health of mice. Though none suffered from dehydration, mice showing signs of dehydration would have been treated with subcutaneous lactated ringers solution. If mice lost greater than 20% of body weight, experienced prolonged dehydration (>1day), or displayed significantly reduced or moribund activity, standard operating procedure was to humanely euthanize prior to experimental endpoint; no mice in our experiments had to be euthanized before the endpoint; however, we did lose 3 total mice (of 39 total hit) immediately after TBI which failed to resuscitate. At the experimental endpoint, mice were sacrificed by deep anesthesia (5% isoflurane), followed by a secondary method of euthanization (for IHC harvest, mice were subject to exsanguination and perfusion fixation with 4% paraformaldehyde in PBS. For vascular edema analysis the brains were removed immediately to be weighed).

### 4.7. Nissl Staining and Cytotoxic Edema Measurements

Brains harvested for IHC were fixed in 4% paraformaldehyde in PBS overnight at 4°C and then were transferred to 30% sucrose in PBS and stored at 4°C for 3 days. Brains were fast-frozen in Tissue-Tek OCT Compound (Sakura, CA) by placing brain molds into 2-methylbutane (Sigma) which had been cooled with liquid N_2_. Coronal brain sections were taken using a cryostat onto gelatin-coated slides and stored at -80°C until use. Frozen coronal brain sections (30 *μ*m) were allowed to dry at room temperature overnight prior to Nissl staining. Nissl staining was achieved after delipidating tissue slices in graduated ethanol washes (70%, 95%, 95%, 100%, and 100%; 2 mins each) followed by a xylenes wash (5 mins). Tissue slices were rehydrated by washing with ethanol in the reverse graduated order (100%, 100%, 95%, 95%, 70%, and 50%; 2 mins) followed by dH_2_O (30 secs). Slides were stained by dipping in 0.13% filtered cresyl violet (Sigma) in H_2_O (30 secs), and then were rinsed in dH_2_O until the water was clear of purple solution. Slides were dehydrated through graduated ethanol washes as before (30 secs) and then washed with xylenes twice (1 min). Slides were allowed to dry and then were mounted using Limonene Mount (Sigma).

Images of cortex and hippocampus near the lesion site were captured via bright-field microscopy and montaged in black and white using a Nikon N-Storm Super-Resolution Microscope equipped with a Photometrics Cool SNAP HQ2 CCD camera. To measure cytotoxic edema, a researcher who was blinded to animal treatments measured cell body size (200-250 cells/mouse on 2 separate brain sections, n=6 mice/group) in the cortex and CA1 region of the hippocampus using ImageJ software (NIH).

### 4.8. Immunohistochemistry

Frozen coronal brain sections (30 *μ*m) were allowed to dry at room temperature overnight, and then heat-mediated antigen retrieval was performed by immersing slides in 0.05% citraconic anhydride pH 7.4 (25 mins) at 95 C and then allowing the slide-containing solution to come to room temperature. Slides were washed twice in PBS (5 mins), incubated in 0.2% triton/PBS (10 mins) and washed once PBS (5 mins). Background autofluorescence was quenched by incubating in 0.1% Sudan Black B/70% ethanol (5 min) and then washed three times in PBS (5 min). Samples were blocked for 1 hr at room temperature with 5% bovine serum albumin/PBS and then were incubated in primary antibody diluted in blocking buffer overnight at 4°C. Samples were washed four times in PBS (15 mins) and incubated in fluorescent-conjugated secondary antibody in blocking buffer for 1 hr at room temperature. Samples were washed once in PBS (5 mins), and nuclei were stained with 4′,6-Diamidino-2-Phenylindole, Dihydrochloride (DAPI, Thermo-Fisher D1306, 1ug/mL)/PBS for 5 mins and then washed twice again in PBS (5 mins). Slides were rinsed in water to remove salt, allowed to dry, and then mounted with Aquapolymount (Polysciences Inc, PA).

### 4.9. Aquaporin Image Analysis

For image capture, researchers were blinded to the animal treatment. Images were captured in the Optical Imaging Core at UT Health Science Center, San Antonio, on a Confocal Ziess LSM710 laser-scanning microscope with a 20X 0.8 NA (Plan-Apo Chromat) objective. Tissue montages of 7 *μ*m z-stacks were captured on the ipsilateral side to the injury and encompassed both the CA1 region of the hippocampus as well as the cortex. Measurement of AQP4 polarization was achieved via methods similar to (20) using FIJI-Image J (NIH): bright perivascular AQP4 was thresholded, and the percentage of pixels within the region-of-interest at or above the mean intensity of perivascular regions was recorded within 500 *μ*m of the lesion area. To normalize data, a control contralateral image was obtained for each brain slice, and ipsilateral values were divided by contralateral values to obtain a ratio. Higher values represent depolarizing movement of AQP4 away from perivascular regions into other astrocytic compartments. To measure GFAP intensity, background was thresholded, and the mean intensity of immunolabeling in the region-of-interest was measured and multiplied by the percentage of pixels above threshold. Values were expressed as a percentage of DMSO-treated sham animal values. For all imaging analysis, individual mouse values were determined from montaged 7 *μ*m z-stacks of 2 separate brain slices. Values were expressed as the mean of n=6 animals/treatment group.

### 4.10. Wet/Dry Brain Weight Measurements

Brain water content was measured with methods similar to previously published protocols [32]. To measure brain water content, the wet weight was measured in freshly harvested brain tissue. Brains were incubated at 37°C in an oven for 4 days to dehydrate the tissue, and then dry weight was measured.

### 4.11. Statistical Analysis

Data are presented as mean +/- one standard error of the mean as calculated by Prism ANOVA analysis followed by Tukey's HSD adjusted for multiple comparisons.* In vivo* experiments included 6 mice for each arm of the experiment. Power analysis was utilized to determine ideal sample sizes needed for power value of 0.8. Statistical significance was taken to be p value < 0.05.

## Data Availability

The data used to support the findings of this study are available from the corresponding author upon request.

## Supplementary Materials

Supplementary Materials Figure S1: Primary Astrocyte Monocultures Uniformly Express the Astrocyte Marker GFAP. Figure S2: AQP4 is Specifically Labeled in Primary Astrocytes. Figure S3: a closer view of AQP4 reorganization in astrocytes after OGD. Figure S4: AQP4 localization does not change in response to astrocyte stretching. Figure S5: cortical Aquaporin 4 isoform expression at 24hrs after TBI.
