# Supplementary material for: Acetazolamide Treatment Prevents Redistribution of Astrocyte Aquaporin 4 after Murine Traumatic Brain Injury
Source: Neurosci J. 2019 May 2;2019:2831501. doi: 10.1155/2019/2831501 (PMC6521570; doi:10.1155/2019/2831501)
Supplement: Supplementary Materials — Figure S1: Primary Astrocyte Monocultures Uniformly Express the Astrocyte Marker GFAP. Figure S2: AQP4 is Specifically Labeled in Primary Astrocytes. Figure S3: a closer view of AQP4 reorganization in astrocytes after OGD. Figure S4: AQP4 localization does not change in response to astrocyte stretching. Figure S5: cortical Aquaporin 4 isoform expression at 24hrs after TBI. [file 2831501.f1.pdf]

Supplementary Figures:

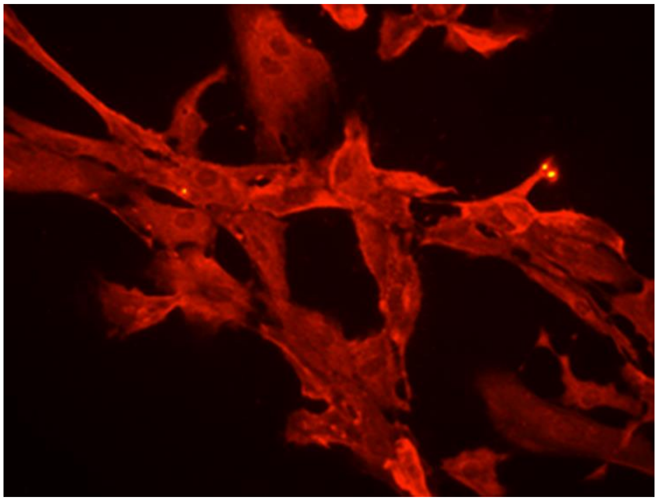

Figure S1: Primary Astrocyte Monocultures Uniformly Express the Astrocyte Marker GFAP. Monocultures were subjected to immunocytochemistry as described in *Methods.* Greater than 90% of cells positively stained for GFAP.

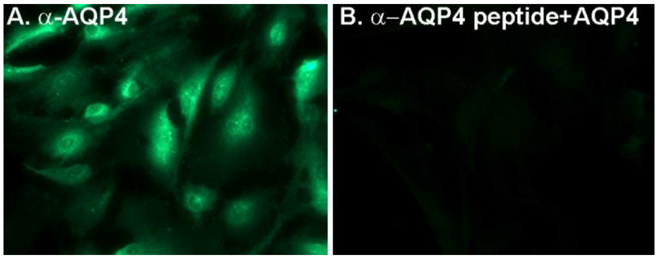

Figure S2: AQP4 is Specifically Labeled in Primary Astrocytes. Fixed astrocytes were pre-incubated with **A)** PBS or **B)** AQP4 blocking peptide before AQP4 immunocytochemistry was performed. Pre-incubation with blocking peptide ablated specific immunolabeling of AQP4 within astrocytes.

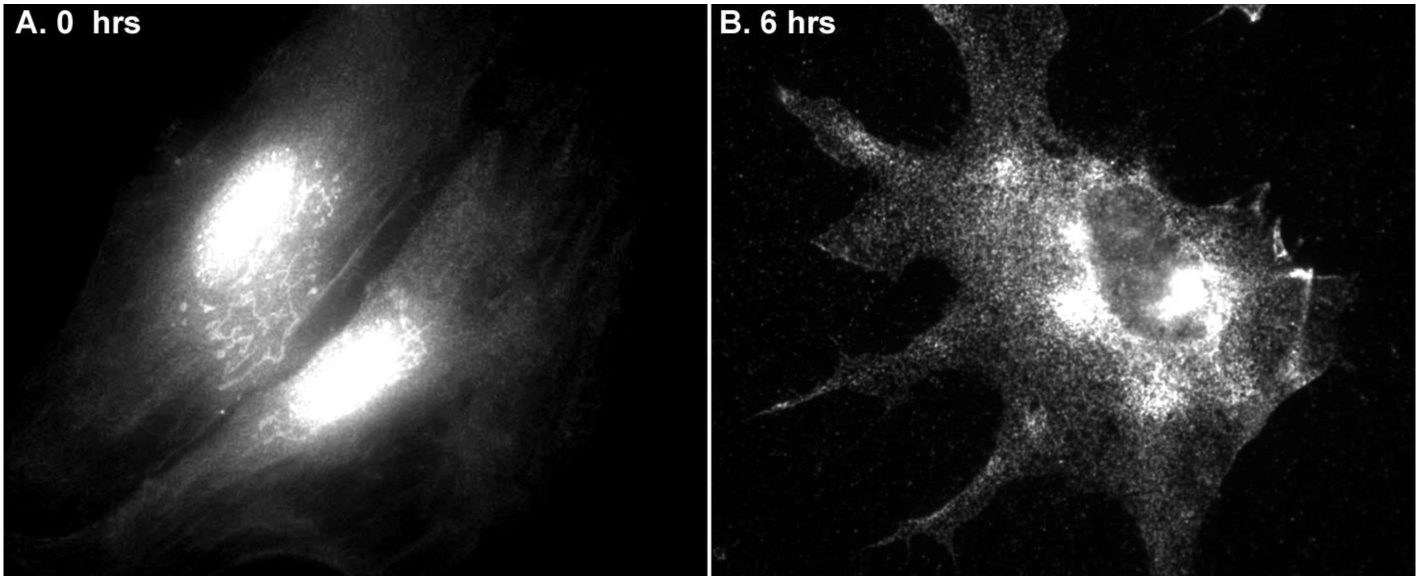

Figure S3: A closer view of AQP4 reorganization in astrocytes after OGD. AQP4 distribution was measured via immunocytochemistry in primary rat astrocytes subjected to **A)** sheer stress alone or **B)** sheer stress plus 6 hrs OGD.

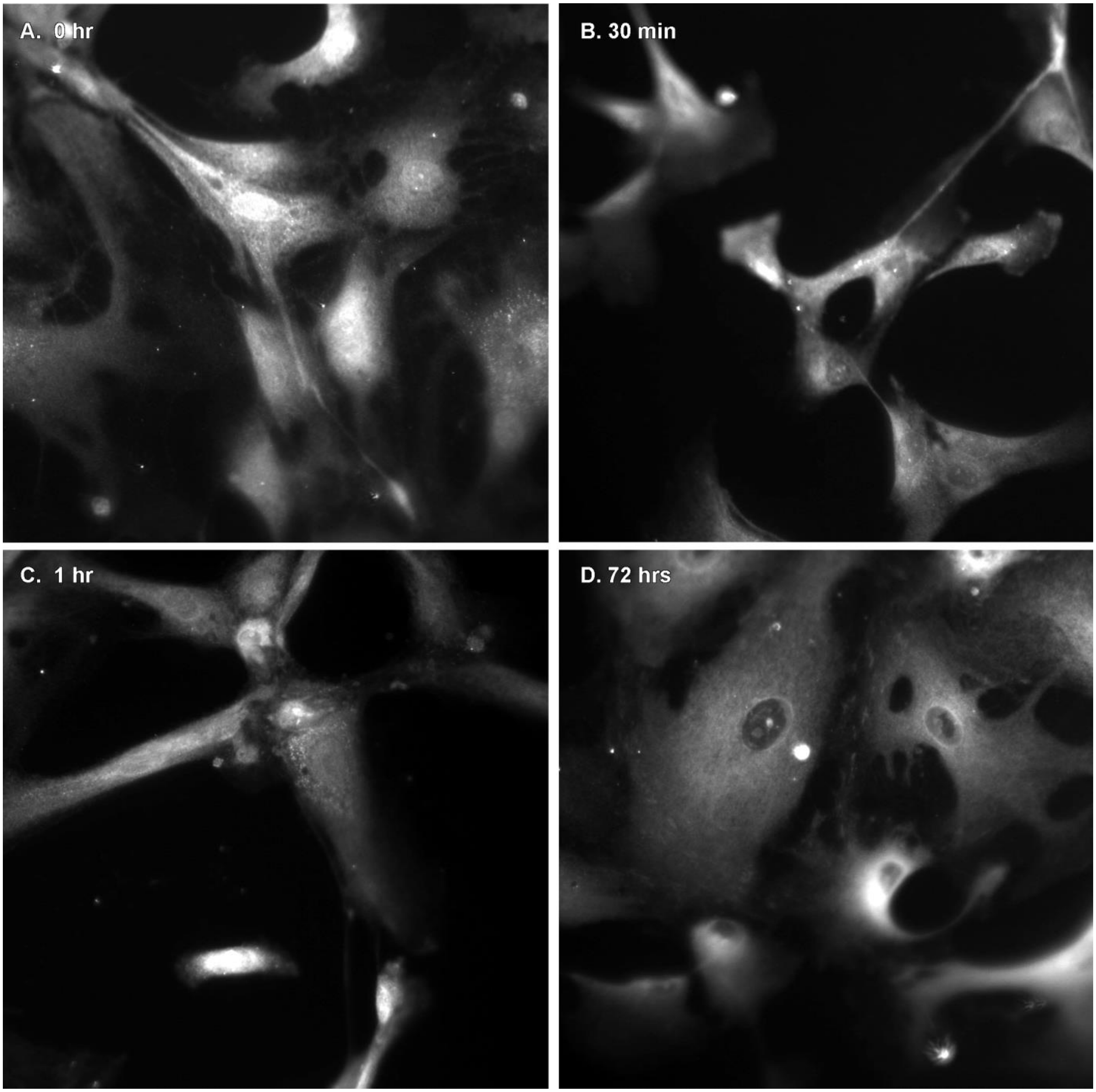

Figure S4: AQP4 Localization Does Not Change in Response to Astrocyte Stretching. Primary astrocytes were subjected to a 5 second stretch to mimic a primary insult after TBI, then were fixed and subjected to immunocytochemistry for AQP4 after **A)** no stretching, **B)** 30 minutes post-stretch, **C)** 1 hour post-stretch, and **D)** 72 hours post stretch.

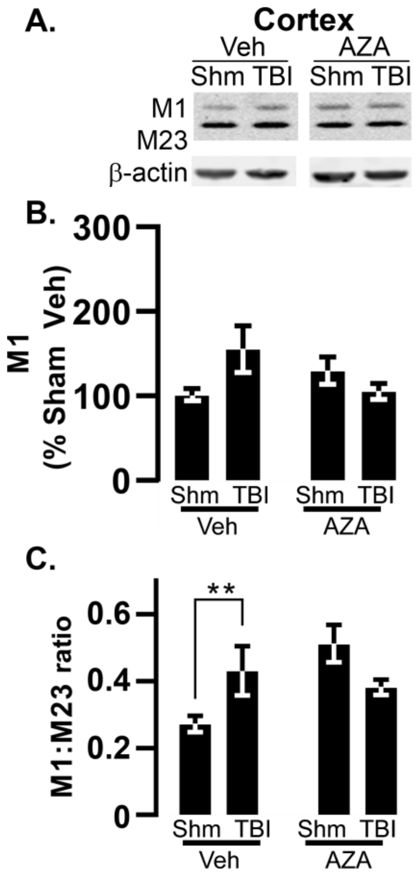

Figure S5: Cortical aquaporin 4 isoform expression at 24hrs after TBI. Mice were subjected to a mild closed-cortical impact, and 30 minutes later were treated with either DMSO (veh) or Acetazolamide (AZA). Tissue microdissection occurred at 24hrs post-TBI. Expression of M1 and M23 were analyzed via SDS-PAGE and western blotting. Results are expressed as means with SEM. ***p<0.01 via ANOVA and Tukey's HSD, n=5 mice/group.*
